# Setting a research agenda for speech therapy and audiology practice in South Africa

**DOI:** 10.4102/sajcd.v72i1.1085

**Published:** 2025-02-28

**Authors:** Katijah Khoza-Shangase

**Affiliations:** 1 Department of Audiology, Faculty of Human and Community Development, University of the Witwatersrand, Johannesburg, South Africa

**Keywords:** research agenda, clinical practice, speech therapy, audiology, South Africa, evidence-based practice, patient-centred care, implementation science

## Abstract

In South Africa, the integration of research into clinical practice within the fields of speech therapy and audiology remains a pivotal, yet underexplored challenge. This opinion piece discusses the critical need for a structured research agenda to drive meaningful changes in clinical practice in South Africa. With healthcare realities such as limited resources, linguistic and cultural diversity, and access to services, it is essential that practitioners, policymakers and researchers collaboratively define and prioritise research topics that address the unique needs of South African patients. Through identifying gaps in current practice, engaging with communities and overcoming barriers to change, the South African healthcare system can adapt to evidence-based, patient-centred models of care. By building a culture of research and innovation, fostering multidisciplinary collaboration and ensuring continuous professional development, South Africa can align its speech therapy and audiology practices with global standards, ultimately improving outcomes for individuals with communication and hearing disorders.

**Contribution:**

This article highlights the steps needed to establish a research-driven framework for clinical practice change, positioning research as a cornerstone of future healthcare delivery.

## Introduction

South Africa is a country rich in linguistic and cultural diversity, where language, hearing and communication disorders impact a significant portion of the population. The role of speech therapists and audiologists in providing critical care to individuals with these conditions is indispensable, but it is clear that the field requires more integration of research into clinical practice (Kathard et al., 2007; Khoza-Shangase et al., 2021). Without a structured approach to research, clinicians are left without the necessary evidence to inform their decision-making, implement innovations or challenge outdated practices. This opinion piece argues that setting a focussed research agenda is crucial for improving the quality of clinical services in speech therapy and audiology, advancing the integration of evidence-based practice and ultimately enhancing patient outcomes in South Africa. The time has come for the speech therapy and audiology professions in South Africa to confront existing challenges and seize the opportunities for improvement through a research-driven agenda. Establishing a formal research agenda is essential for clinicians to stay abreast of global innovations while ensuring local contextual relevancy. However, critics might argue that adopting a global research framework could save resources and align South African practices with international standards. While this perspective highlights the efficiency of universal models, it risks overshadowing local relevance and context.

## The need for a research agenda: A call for change

Research agendas act as a roadmap for addressing clinical challenges, shaping practice and guiding innovation. A robust research agenda provides a clear path for healthcare professionals to follow, ensuring that research efforts are not random or fragmented but are strategically aimed at solving the problems that matter most to both practitioners and patients (Olatunji et al., 2023; World Health Organization, 2023). In South Africa, healthcare delivery in speech therapy and audiology faces several unique realities, including a diverse linguistic landscape, disparities in access to care and systemic resource constraints (Khoza-Shangase, 2021; Pillay et al., 2020). These challenges create the need for a more focussed and localised approach to research, one that speaks directly to the issues faced by South African populations. While the argument for localised research is compelling, it is important to acknowledge the potential advantages of externally derived research. For instance, global research initiatives often provide access to extensive funding, tested methodologies and international collaboration. However, these benefits must be weighed against the risks of adopting models that fail to consider local cultural, linguistic and socio-economic factors, potentially rendering interventions less effective or inappropriate for the South African context.

Expanding a research agenda to address issues faced by South African populations means targeting studies and clinical efforts that consider the country’s unique socio-economic and cultural context. South Africa’s healthcare landscape is characterised by its rich linguistic diversity – 11 official languages and many regional dialects – which affects the accessibility and effectiveness of speech therapy and audiology services (Magaqa, 2021). For instance, language differences often hinder accurate diagnosis and culturally relevant treatment for patients who may not speak English and/or Afrikaans as a primary language (Khoza-Shangase & Mophosho, 2018; Mdlalo et al., 2019). Moreover, the disparities in access to care between urban and rural areas pose additional challenges, with rural populations often lacking adequate healthcare infrastructure and skilled professionals. This urban-rural divide impacts the timeliness and quality of interventions, leading to preventable delays in treatment and poorer health outcomes (Mkhize et al., 2022). In response, a research agenda ‘that speaks directly to the issues faced by South African populations’ should prioritise studies on multilingual diagnostic tools, culturally competent interventions and scalable service delivery models that accommodate rural areas, such as telehealth. By developing evidence-based strategies rooted in South Africa’s unique needs, such a research agenda can lead to more equitable healthcare delivery, ultimately improving the quality of life for individuals across the nation.

A formalised research agenda would not only direct research efforts but would also provide a foundation for improving clinical practice and patient care. Without this focus, clinicians may continue to rely on research outcomes developed in different cultural and socio-economic contexts, which may not always be appropriate for the South African context. For example, therapeutic models designed for affluent, English-speaking populations may not work as well for the multilingual communities in South Africa, where socio-economic disparities exacerbate health inequities, and where health seeking and treatment adherence behaviours may be vastly different.

## Identifying key areas for research in speech therapy and audiology

In the South African context, there are several areas where research efforts could make an immediate and meaningful impact. The key areas highlighted in this article were identified through a combination of approaches, including a review of relevant literature, analysis of existing gaps in the South African speech-language pathology and audiology (SLP/A) landscape and professional experience within the field. The selection process also drew on global trends in SLP/A research, contextualised to address South Africa’s unique linguistic, cultural and socio-economic realities. These areas reflect both the priorities identified in the literature and the pressing needs observed in clinical practice:

1. *Evidence-based practice*: As speech therapy and audiology become increasingly evidence-driven globally, South Africa must not lag in incorporating best practices based on local evidence (Kathard et al., 2007; Moodie et al., 2011). Developing research focussed on the efficacy of specific therapies for diverse linguistic and cultural groups will improve clinical interventions, ensuring they meet the needs of a South African population that is multilingual, multiethnic and socioeconomically varied. A potential counterargument to prioritising localised evidence is the resource-intensive nature of developing region-specific research. With limited funding and a shortage of professionals, critics might argue that adapting existing global evidence could be a more practical solution. While this approach could streamline implementation, it may inadvertently perpetuate inequities by failing to address the unique challenges faced by South African populations.
2. *Patient-centred care*: Speech therapists and audiologists should prioritise patient-centred care approaches (Mahomed-Asmail et al., 2024), but research is needed to determine the most effective ways to implement these practices in South Africa’s public healthcare system. Studies exploring the perspectives of patients and their families will help tailor interventions to be more individualised and culturally sensitive. Research that investigates patient experiences with service delivery in rural versus urban settings could also provide valuable insights into overcoming disparities.
3. *Innovative service delivery models*: South Africa’s healthcare system faces resource limitations, with a well-documented capacity versus demand challenge around speech therapy and audiology workforce, making innovative service delivery models a necessity (Pillay et al., 2020). Research into telehealth, mobile health and outreach clinics could help bridge the gap between urban and rural care, as well as face-to-face and remote interventions. These models could potentially expand access to care, especially for patients in underserved or remote areas (Swanepoel, 2023). Furthermore, studies on the feasibility, effectiveness and sustainability of such models could inform policy decisions and set the stage for nationwide adoption.
4. *Technological integration*: Advances in technology offer significant potential in both diagnostics and treatment (Brice et al., 2024; Frosolini et al., 2024; Maluleke & Khoza-Shangase, 2023). The integration of assistive technologies, mobile apps for therapy and telemedicine into the practice of speech therapy and audiology could vastly improve the quality and accessibility of care. South African research into how these technologies can be applied in local settings will be key to ensuring they are effective and beneficial for patients. Research into the use of AI in diagnostic tools or rehabilitation could also revolutionise how hearing and communication disorders are addressed in the country.

Table 1 provides additional research areas. These additional research areas would provide a comprehensive framework for advancing speech and hearing healthcare in South Africa. The prioritisation of these areas is based on an iterative review of the literature, alignment with global trends in speech and audiology research, and insights drawn from professional experience within the South African context. This ensures that the proposed interventions are not only clinically effective but also culturally relevant, accessible and sustainable across different populations. Future studies could further refine and expand these priorities by engaging a broader range of stakeholders, including policymakers, community members and interdisciplinary professionals. While these research areas highlight where efforts should be focussed, effectively addressing them will require overcoming significant barriers and ensuring that research findings are seamlessly integrated into clinical practice. These priorities are closely tied to the need for strategies that address systemic challenges, foster community engagement and establish pathways for translating research into actionable interventions. The following sections discuss how these processes can support the proposed research areas and ensure their real-world impact.

**TABLE 1:** Additional research areas that could be part of the research agenda.

Research Area	Focus description
Language and cultural adaptation of assessments and interventions	Research is needed on adapting diagnostic tools and therapeutic approaches to South Africa’s multilingual and multicultural setting. This includes developing standardised tests and therapies that are valid across diverse linguistic and cultural groups.
Health literacy and community awareness	Examining ways to increase public understanding of communication disorders is critical, especially in underserved areas. Research can explore how best to educate communities on early detection and intervention and raise awareness of available resources.
Early identification and intervention models	Focus on strategies for identifying speech, language and hearing issues early in life, particularly in rural and lower-income areas where resources are limited. Studies can explore feasible models for screenings in schools, clinics or through mobile health units.
Workforce development and training	Investigate methods for training more speech-language therapists and audiologists, particularly in rural or underserved areas. This may include exploring alternative pathways, task-sharing models with community health workers, and continuous professional development in emerging areas.
Social determinants of health in communication disorders	Research on how factors like socio-economic status, education, housing and nutrition influence the prevalence and impact of communication disorders could inform targeted interventions that address these underlying determinants.
Tele-audiology and teletherapy	Further studies on the effectiveness, accessibility and challenges of telehealth solutions in speech and hearing care, with an emphasis on connectivity issues and technology adaptation for rural or resource-limited settings, could significantly impact service delivery.
Mental health and communication disorders	Explore the intersection between communication disorders and mental health issues, such as anxiety and depression, particularly in children with communication challenges and in adults with hearing loss, to guide holistic and supportive care practices.
Ageing population and hearing care	With a growing elderly population, research on age-related hearing loss, cognitive decline and the accessibility of hearing care services could lead to better support and intervention frameworks for older adults.
Inclusive education and communication disorders	Research focussing on effective integration of children with communication disorders in mainstream schools, including support for teachers, training in communication techniques and policies to facilitate inclusion, could help promote equity in education.
Economic evaluation of speech and hearing services	Conducting studies on the cost-effectiveness and long-term economic impact of early interventions and regular hearing care can support advocacy efforts for funding and resource allocation in these areas.

In compiling the above-stated research areas, priority was given to topics frequently emphasised in peer-reviewed studies, as well as areas that have shown significant potential for impact based on local clinical experiences and community feedback. The inclusion of these areas is not exhaustive but highlights the most urgent research priorities for advancing speech and hearing care in South Africa.

## Overcoming barriers to change in the South African context

Achieving progress in the identified research areas requires addressing systemic barriers that hinder the integration of research into clinical practice. These barriers are not unique to South Africa but are especially pronounced in the country because of its unique socio-political and economic challenges. These barriers – ranging from resource limitations to resistance to change – impact the ability to implement interventions that stem from research findings. By overcoming these barriers, South African speech therapy and audiology professionals can translate the proposed research areas into practical, contextually relevant solutions that improve patient outcomes.

1. *Resistance to change*: Resistance to new practices is often rooted in a lack of awareness or understanding. Clinicians who have practised for years in a traditional manner may be hesitant to adopt new research-driven practices, especially if they are unfamiliar with them or feel inadequately trained (Maluleke & Khoza-Shangase, 2023). Overcoming this resistance requires leadership from professional organisations, as well as a concerted effort to integrate evidence-based practices into both training programmes and clinical settings. It is also worth noting that resistance to change may sometimes stem from legitimate concerns about the feasibility and sustainability of implementing localised research findings. Clinicians might worry about the time and resources required to adapt their practices, particularly when existing models derived from global research appear to work adequately. Addressing these concerns requires a balanced approach that integrates both localised adaptations and evidence from broader contexts.
2. *Limited resources and funding*: The South African healthcare system, particularly in the public sector, suffers from significant resource limitations, including inadequate funding for research, training and infrastructure. Researchers often face financial challenges in conducting studies, and healthcare professionals struggle to access the latest research findings (Magaqa, 2021; Moodie et al., 2011; World Health Organization, 2023). The lack of dedicated funding for speech therapy and audiology research limits the ability to conduct studies tailored to South Africa’s unique needs. While governmental bodies like the National Research Foundation (NRF) and the Department of Health (DoH) could play a role in funding these initiatives, partnerships with private sector organisations, philanthropic foundations and international donors must also be explored. In addition, there is limited collaboration between academic institutions and public healthcare facilities, which could serve as research sites for pilot studies and implementation trials. Bridging these gaps will require active engagement with policymakers to integrate research priorities into national and provincial healthcare strategies. In addition, addressing this requires advocacy for increased funding for clinical research in the speech therapy and audiology fields, both from government bodies and private sectors.
3. *Cultural and institutional resistance*: There is also resistance within institutions to change, particularly when research findings require modifications to established practices (Bhattacherjee & Hikmet, 2007). Institutions must be willing to embrace research as a critical tool for change and improvement. This will require buy-in from institutional leadership, as well as a shift in organisational culture towards valuing continuous learning and evidence-based practices.
4. *Shortage of specialised professionals*: South Africa faces a shortage of trained speech-language therapists and audiologists, especially in rural and underserved areas (Pillay et al., 2020). This shortage limits access to services and slows the implementation of new research findings, as fewer professionals are available to champion change and adopt innovative practices. Addressing this barrier requires investment in workforce development, task-sharing models and incentives for professionals to work in areas with the greatest need.
5. *Limited access to reliable data and research resources*: Access to up-to-date research, reliable health data and clinical resources can be limited in many South African healthcare settings, especially outside urban centres (Seahloli, 2017). This gap restricts practitioners’ ability to stay informed about best practices, new technologies and evidence-based methods, hampering efforts to incorporate research findings into practice. Building a more accessible national database of health information and improving digital access to research resources would help mitigate this barrier.

## The role of community and stakeholder engagement

Community and stakeholder engagement are integral to addressing the research areas identified earlier, particularly in creating culturally relevant and patient-centred care models. Engaging with communities not only ensures that research priorities align with real-world needs but also fosters trust and collaboration, both of which are crucial for implementing sustainable changes in practice. For example, research into telehealth solutions or multilingual diagnostic tools must be guided by insights from the communities they aim to serve. For example, communities in rural areas may prioritise research into mobile hearing screenings or telehealth services that are more accessible than traditional in-person consultations. In addition, collaboration across various disciplines – such as educators, psychologists, social workers, healthcare policymakers and traditional healers – can enhance the value of research findings and create a holistic approach to patient care. South Africa’s interdisciplinary approach can ensure that research translates into more effective service delivery models, better outcomes for patients, and a more integrated healthcare system.

## Implementing research into practice

The ultimate goal of addressing the proposed research areas is to bridge the gap between theory and practice, ensuring that findings lead to actionable improvements in clinical care. Translational research plays a crucial role in turning research into practice by creating evidence-based guidelines and training programmes that directly respond to the priorities identified earlier. For instance, a research focus on innovative service delivery models, such as telehealth, must be complemented by implementation strategies that consider infrastructure challenges and workforce readiness. In the South African context, this involves addressing the logistical and systemic challenges that often impede implementation. For example, evidence-based clinical guidelines must be developed collaboratively with input from practitioners, researchers and policymakers to ensure their applicability in diverse settings. Capacity-building initiatives, such as workshops and training sessions led by South African Speech-Language-Hearing Association (SASLHA) or South African Association of Audiologists (SAAA) or academic institutions, can equip clinicians with the skills to incorporate research findings into practice. In addition, leveraging digital platforms for knowledge dissemination, such as online repositories of evidence-based resources, can overcome geographical and resource-related barriers. Translational research is key to ensuring that scientific findings do not remain confined to journals but are translated into actionable strategies that benefit patients (Ioachimescu & Shaker, 2024; Olatunji et al., 2023). This process requires clear communication, the development of clinical guidelines based on the latest research and a commitment to continuous professional development for practitioners. The role of professional associations and regulatory bodies is critical in this process. They should not only advocate for research but also ensure that clinical guidelines are developed and updated regularly to reflect the latest evidence. Furthermore, providing training and resources for clinicians to integrate new research into their practice is essential for long-term success.

## Conclusion: Setting a future research agenda

The call for a research agenda in speech therapy and audiology in South Africa is not only an academic exercise but a practical necessity. The country’s diverse linguistic and cultural landscape, coupled with disparities in healthcare access and resource constraints, requires a research agenda tailored to local needs. Currently, much of the research influencing clinical practice in South Africa originates from high-income countries, whose socio-economic realities differ significantly. While international evidence has value, it must be adapted to the local context to be effective. Establishing such an agenda will require coordinated efforts involving academic institutions, professional bodies like SAAA, SASLHA, healthcare policymakers and community representatives. These stakeholders are uniquely positioned to identify priorities, secure funding, and drive implementation. As the country navigates complex healthcare challenges, it is crucial that speech therapy and audiology professionals actively contribute to shaping the future of their professions. However, it is important to acknowledge the challenges and criticisms that such an approach might face. Critics may argue that resource constraints, including funding and workforce shortages, make a localised research agenda difficult to sustain. Others may point out the risk of overemphasising localised research to the detriment of leveraging well-established global evidence. Despite these challenges, the proposed approach remains transformative because it provides an opportunity to address the unique needs of South African populations in a way that globally derived models often cannot. A locally relevant, evidence-driven and community-centred research agenda ensures that interventions are not only scientifically valid but also culturally, linguistically, and socially appropriate. Furthermore, integrating international perspectives into localised research can balance resource constraints by adapting proven methodologies to the South African context. Setting a research agenda that is locally relevant, evidence-driven and community-centred will pave the way for clinical practices that are more effective, efficient, and equitable. This article advocates for localised research as a foundation for advancing clinical practice. However, it is equally important to consider how global research can complement these efforts. By integrating international insights into a localised framework, South African speech therapy and audiology can balance efficiency and relevance, ensuring a more comprehensive approach to healthcare delivery. While the road ahead may be complex for the speech therapy and audiology professions in South Africa, the transformative potential of a research agenda grounded in South African realities lies in its ability to foster a culture of research, innovation and collaboration, and to encourage both seasoned professionals and young practitioners to contribute to this vital work.
